# Effects of unilateral versus bilateral training on linear sprint and change-of-direction performance in athletes: a systematic review and meta-analysis

**DOI:** 10.3389/fphys.2026.1884017

**Published:** 2026-08-17

**Authors:** Chen Dong, Yugang Zhang, Min Sun

**Affiliations:** 1 School of Physical Education, Xi’an Petroleum University Xi’an, Shaanxi, China; 2 Department of Physical Education, Yuncheng University, Yuncheng, China; 3 School of Physical Education, Baoshan University, Baoshan, Yunnan, China

**Keywords:** acceleration, athletes, COD ability, neuromuscular training, sprint performance

## Abstract

**Background:**

Unilateral training may enhance sprint and change-of-direction (COD) performance in athletes, but existing evidence is inconclusive.

**Methods:**

We searched CNKI, PubMed, Web of Science, and EBSCO up to 1 September 2025 for randomized controlled trials comparing unilateral versus bilateral training in athletes and reporting speed-related outcomes. Methodological quality was assessed with PEDro. Random-effects meta-analyses computed mean differences (MD), and GRADE was used for certainty grading.

**Results:**

Fourteen studies (415 male athletes, aged 9–27 years) were included. Unilateral training showed no significant pooled effects on 10 m, 20-m, or 30 m sprints (overall *I²* = 58%), the T-test (*I²* = 59%), or COD to the right (COD-R, *I²* = 44%). The 505 test was not pooled because of very high heterogeneity (*I²* = 92%) and conflicting individual findings. Significant improvements favoring unilateral training were observed for 5 m sprint (Z = 2.01, *P* = 0.04) and COD to the left (COD-L, Z = 2.13, *P* = 0.03). Age subgroup analysis (<18 vs. ≥18 years) did not moderate 10 m sprint results (*P* = 0.64). Overall evidence quality was low to moderate.

**Conclusion:**

Unilateral training confers selective, small-magnitude benefits for 5 m sprint and COD-L, but these results are preliminary. The COD-L finding rests on only three studies (63 athletes), and the 5-m finding on four studies (113 athletes). Given the exclusively male sample and wide age range (9–27 years), generalization requires caution. Unilateral training did not universally improve all sprint or COD measures. While results for 30-m sprint, T-test, and COD-R were relatively consistent, the 505-test evidence should be interpreted with caution because of high heterogeneity. Future large-scale RCTs should explore applicability across sports, age groups, and sexes, with attention to maturational status and training age in younger populations.

**Systematic Review Registration:**

https://www.crd.york.ac.uk/prospero/display_record.php?RecordID=1031255, identifier CRD420251031255.

## Introduction

Athletes’ outstanding performance is closely related to physical performance capacity, technical–tactical execution ability, and psychological factors (Smith, 2003). Among these determinants, physical performance capacity is widely regarded as a key factor influencing athletic level and competitive success (Gabbett et al., 2007; Xiao et al., 2021). Within physical fitness components, linear sprint and change-of-direction (COD) speed are considered fundamental for sustaining athletic performance and serves as a basis for coping with high-intensity and high-complexity competitive demands (Tingelstad et al., 2023). In practice, deficiencies in these speed qualities have been associated with lower competitive performance and a higher risk of sports-related injuries (Farley et al., 2020). For example, in field hockey, superior linear sprint and COD abilities provides athletes with more time to execute technical skills and reduces injury risk during rapid deceleration and change-of-direction movements (Malone et al., 2019).

Empirical evidence has demonstrated that training interventions can improve athletes’ linear sprint and COD performance. The American College of Sports Medicine recommends a progressive approach combining load progression and speed training to enhance these physical qualities (Bishop et al., 2024). However, other studies have reported that such combined approaches may have limited transferability to sport-specific performance (Blazevich and Jenkins, 2002). This may be explained by the fact that a single sport skill requires the coordinated contribution of multiple joints, muscle groups, and multidirectional speed components (Wulf et al., 2010). In other words, athletic performance in competition emerges from the integration of multi-joint coordination, muscular synergy, and multidirectional speed expression. Therefore, training models that target a single physical attribute may be insufficient to substantially enhance overall athletic performance. To improve training effectiveness, a shift from “single-attribute development” toward “movement pattern integration” is required.

Against this background, unilateral training has received increasing research attention regarding its effects on athletes’ physical performance (Zhang et al., 2023). Compared with bilateral training, unilateral training may enhance core stability and motor control through unilateral strength, sport-specific skill, and speed-oriented training stimuli (Duong et al., 2022; Waller et al., 2008).

Several empirical studies have shown that unilateral training positively affects strength (Lee et al., 2021), explosive power (Makaruk et al., 2011), and agility (Escobar Hincapie et al., 2021). In addition, positive effects have also been reported in soccer players (Ramirez-Campillo et al., 2018) and rugby players (Speirs et al., 2016). Despite these beneficial effects across multiple physical fitness domains, there is still a lack of systematic synthesis comparing unilateral training-based programs with bilateral training regarding their effects on athletes’ linear sprint and COD performance.

Conceptually, unilateral training refers to exercises performed with a single limb, using asymmetrical stimuli to promote muscular development and inter-limb balance in terms of strength, flexibility, and technical control (Taniguchi, 1997). Compared with bilateral training, unilateral training requires athletes to perform coordinated multi-joint movements under unilateral support or force production while maintaining postural stability (Kassiano et al., 2025). This training modality involves coordinated activation of multiple joints, such as the hip and knee, and aligns with the biomechanical demands of running, acceleration, and change-of-direction tasks characterized by unilateral force production (Appleby et al., 2019). Compared with bilateral training, unilateral training places greater emphasis on unilateral strength and neuromuscular control, which may in turn contribute to improved inter-limb balance (Thorpe and Ebersole, 2008). Accordingly, unilateral training is often considered to enhance linear sprint and COD performance by improving inter-limb balance (Zhang et al., 2026). Some empirical evidence has suggested that unilateral training may confer advantages over bilateral compound training in improving short-distance sprint performance (Fisher and Wallin, 2014). However, most existing studies are based on small sample sizes, and heterogeneity in training methods, intervention duration, and frequency may have contributed to the inconsistent findings reported across studies. In addition, most quantitative syntheses focusing on unilateral training and speed-related outcomes have examined specific training modalities, such as unilateral resistance training, rather than systematically comparing unilateral and bilateral training effects (Liao et al., 2022). Consequently, systematic evidence that integrates the effects of different unilateral training modalities remains limited.

Therefore, this study employs a meta-analytic approach to synthesize existing empirical evidence, evaluate the effects of unilateral training compared with bilateral training on athletes’ linear sprint performance (across 5 m, 10 m, 20 m, and 30 m) and COD performance (including T-test, 505 test, and unilateral 505-L/R tests), and further explore how different unilateral training protocols and intervention characteristics influence training outcomes. The findings aim to provide evidence-based support for athletic training practice.

## Methodology

### Protocol and registration

This systematic review and meta-analysis was conducted in accordance with the Preferred Reporting Items for Systematic Reviews and Meta-Analyses (PRISMA) guidelines (Moher et al., 2009). The study protocol was prospectively registered in the International Prospective Register of Systematic Reviews (PROSPERO) on 12 April 2025 (registration number: CRD420251031255; available at: https://www.crd.york.ac.uk/prospero/display_record.php?RecordID=1031255). The review and analyses were performed in full accordance with the registered protocol, with no deviations.

### Search strategy and selection process

The initial literature search was conducted on September 1, 2025, across four databases: China National Knowledge Infrastructure (CNKI), PubMed, Web of Science, and EBSCO. To include recently published studies, we performed an updated search on March 15, 2026, using the same databases and search strategy. The development of search terms and free-text keywords was based on previous systematic reviews (Zhang et al., 2024; Tao et al., 2025), which informed the construction of the search strategy. In each database, title-based searches were performed using Boolean operators. The following search terms and combinations were used: (Athletes OR athlete* OR player*) AND (Unilateral training OR Single-leg training OR Unilateral exercise OR Single-limb training OR Asymmetrical training) AND (Linear sprint OR Sprint performance OR Change of direction OR COD OR Agility OR Speed OR Acceleration). In addition, a manual search was conducted in Google Scholar, including screening the reference lists of relevant articles and performing supplementary keyword searches to identify additional eligible studies. Furthermore, the reference lists of all included studies were reviewed to identify any potentially missing publications. Finally, experienced library information specialists were consulted to support and optimize the data retrieval process.

### Eligibility criteria

The inclusion criteria for this study were established based on the PICOS framework (Population, Intervention, Comparison, Outcomes, and Study Design), as detailed in **Table 1**. Only studies examining the effects of unilateral training on athletes’ linear sprint and/or change-of-direction (COD) performance were considered eligible. Accordingly, studies were included if they met the following criteria:

**Table 1:** Inclusion criteria based on the PICOS framework.

Item	Inclusion criteria
Population	Athletes (male/female)
Intervention	unilateral training
Comparison	Bilateral training or control group
Outcome	Movement Speed
Study designs	Randomized controlled trial (RCT)

1. Studies published in Chinese or English and subjected to peer review were included. The participants were male or female athletes, and the studies aimed to investigate the effects of unilateral training on athletes’ linear sprint and/or COD performance. Eligible studies were required to adopt a randomized controlled trial design with at least two groups.
2. Included studies were required to implement planned and structured unilateral training interventions designed to improve athletes’ linear sprint and/or COD performance. Studies combining unilateral training with other training modalities (e.g., unilateral plyometric training) were also considered eligible.
3. Included studies had to examine the effects of unilateral training on athletes’ linear sprint and/or COD performance and report at least one outcome measure related to these physical qualities.
4. There were no restrictions on the type of sport, age, or sex of the athletes. Additionally, no limitations were imposed on sample size, study location, or intervention duration.

Studies meeting the following criteria were excluded:

1. Studies that incorporated non-training interventions in addition to unilateral training (e.g., psychological interventions), as well as studies without supervised training, were excluded.
2. Studies published in languages other than Chinese or English, as well as theses and conference proceedings, were excluded.
3. Studies that did not employ a randomized controlled trial design were excluded.

### Data extraction

Two authors (CD and SM) independently extracted data from the included studies using Microsoft Excel spreadsheets, and a third author (ZYG) reviewed the extracted information for accuracy. The extracted data included: (a) first author, year of publication, and country; (b) participant characteristics, including age, sport type, sex, training experience, performance level, and sample size; (c) details of the unilateral training intervention, including duration, frequency, and training type; (d) Assessment of sprint and change-of-direction (COD) performance: For linear sprints, we extracted the testing distance (e.g., 5 m, 10 m, 20 m, 30 m) and timing method. For COD tests (e.g., T-test, 505 test, COD-L, COD-R), we extracted: (d1) the spatial definition of left and right directions (e.g., defined relative to the participant’s own sagittal plane or to absolute external markers); (d2) the specific cutting angle (e.g., 45°, 90°, or 180° turn); and (d3) the timing start/trigger mechanisms (e.g., photocells, timing gates, or manual stopwatch); (e) Laterality and biomechanical context of COD tasks: To interpret directional asymmetries, we extracted the following variables from each original study: (e1) the criteria used to determine the dominant leg (e.g., preferred kicking leg, unilateral jump distance, or force production); (e2) the described roles of the support (stance/braking) leg and swing leg during the cutting maneuver; (e3) the testing order of COD-L and COD-R (e.g., randomized, counterbalanced, or fixed sequence) and whether adequate rest intervals were provided between directions; and (e4) whether the authors considered sport-specific directional demands (e.g., left/right turn preferences in match play); (f) the specific method used to equate training volume between the unilateral and bilateral groups (e.g., based on total repetitions, repetitions per limb, total load, mechanical work, total training time, or relative intensity), as this factor was a primary source of methodological heterogeneity considered in our descriptive analysis; and (h) Management of unreported data: Any item listed in (d) or (e) that was not clearly reported in the original study was coded as “not reported (NR)” in our extraction spreadsheet. The overall completeness of reporting regarding directional definitions and testing order was descriptively summarized, and this summary was used to contextualize the observed heterogeneity in our subgroup analyses.

### Study quality assessment

Two reviewers (CD and SM) independently assessed the methodological quality of the included studies using the Physiotherapy Evidence Database (PEDro) scale, a tool that has been demonstrated to be valid (De Morton, 2009) and reliable (Maher et al., 2003). The PEDro scale comprises 11 methodological criteria; each satisfied criterion contributes one point to the total score, with Item 1 excluded from the final quality score calculation. The assessment results were cross-checked by a third reviewer (ZYG), and any disagreements were resolved through discussion until consensus was reached among all three reviewers. The total PEDro score ranges from 1 to 10, with scores ≤ 3 indicating poor methodological quality, scores of 4–5 indicating moderate quality, and scores of 6–10 indicating high methodological quality. Studies with a PEDro score below 3 were excluded prior to the certainty of evidence evaluation to minimize the risk of moderate to high bias.

### Risk of bias assessment

Two reviewers (CD and SM) independently assessed the risk of bias for each included study using the original Cochrane Risk of Bias tool (Version 1.0). The following seven standard domains were evaluated: random sequence generation, allocation concealment, blinding of participants and personnel, blinding of outcome assessment, incomplete outcome data, selective reporting, and other bias. Each domain was judged as ‘low risk,’ ‘unclear risk,’ or ‘high risk’. For COD-specific outcomes, we additionally evaluated whether the testing order for COD-L and COD-R was randomized (to avoid fatigue bias) and whether both directions were reported separately (rather than as a composite score). Disagreements were resolved by discussion with a third reviewer (ZYG). For outcomes with very few studies, RoB results were interpreted with particular caution. The results of this RoB assessment were subsequently used to inform the ‘risk of bias’ domain in the GRADE evaluation.

### Certainty of evidence

Two reviewers (CD and SM) independently assessed the certainty of evidence for the included studies using the Grading of Recommendations Assessment, Development and Evaluation (GRADE) approach, classifying the evidence into four levels: very low, low, moderate, and high (Zhang et al., 2019). Prior to assessment, all outcome indicators were initially considered to be of high certainty. The evidence level for each outcome was then downgraded according to the following criteria:

a. Risk of bias: downgraded by one level if the original Cochrane Risk of Bias tool (Version 1.0) assessment indicated ‘high risk’ in one or more critical domains (e.g., randomization process or allocation concealment), or by two levels if ‘high risk’ was identified in multiple critical domains.
b. Indirectness: because study selection strictly followed PICOS criteria, ensuring alignment between participants, interventions, and outcomes, the risk of indirectness was considered low (no downgrading).
c. Publication bias: downgraded by one level when reliable statistical evidence of publication bias was detected. However, for most outcomes in this study, the number of included studies was fewer than 10, which precludes the reliable use of funnel plot asymmetry tests or Egger’s regression test. Therefore, publication bias was not routinely downgraded unless funnel plot asymmetry was clearly evident and the outcome included 10 or more studies with a statistically significant Egger’s test (P<0.05) as a complementary evidence. For outcomes with fewer than 10 studies, funnel plots were visually inspected but interpreted with caution, and no downgrading for publication bias was applied due to the limited power of detection methods.
d. Inconsistency: downgraded by one level when statistical heterogeneity (I²) exceeded 75%.
e. Imprecision: downgraded by one level when the optimal information size was not met and the confidence intervals overlapped the null effect; if both conditions were present, two levels were downgraded. For outcomes that could not be pooled in a meta-analysis, the certainty of evidence was downgraded by two levels due to very serious imprecision (limited sample size and wide confidence intervals), resulting in a rating of very low certainty.

### Statistical analysis

Although a meta-analysis can technically be conducted with as few as two studies (Valentine et al., 2010), sample sizes in sports science research are generally small. Therefore, in the present study, meta-analyses were only performed when at least three studies were available. Meta-analyses for COD-L and COD-R were performed separately as independent outcomes. To compute the mean differences (MD) between groups, post-intervention means and standard deviations were extracted for each outcome. In cases where post-intervention data were not directly available, change scores from baseline were used instead, and the variance of change scores was calculated using an assumed correlation coefficient of r = 0.7, based on previous meta-analyses in sports science (Deng et al., 2023). Sensitivity analyses were subsequently conducted using r=0.5 and r=0.8 to test the robustness of our findings. When required data were not directly available, corresponding authors were contacted to obtain the original datasets. In cases where data were presented only in graphical form, values were extracted using WebPlotDigitizer (version 4.5; https://apps.automeris.io/wpd/) (Drevon et al., 2017).

Meta-analyses were conducted using a random-effects model with inverse-variance weighting, where each study was weighted according to its standard error (Deeks et al., 2019), thereby improving the robustness of the pooled estimates. Given the potential heterogeneity across interventions and participant populations among the included studies, the random-effects model was prespecified as the primary analytical framework for all pooled effect estimates, whereas the fixed-effect model was used only as a supplementary tool for sensitivity analyses. Pooled mean differences (MD) were reported with 95% confidence intervals. When a study included multiple intervention groups, the control group was proportionally allocated to avoid unit-of-analysis errors and to ensure independent comparisons across groups (Deng et al., 2023). Heterogeneity was assessed using the I² statistic, with thresholds defined as <25% (low), 25–75% (moderate), and >75% (high heterogeneity) (Higgins and Thompson, 2002). Accordingly, we prespecified that outcomes with I²>75% would not be quantitatively pooled; instead, they would be summarized narratively. Given the unadjusted multiple comparisons, all p-values are considered exploratory; significant findings (p < 0.05) should be interpreted cautiously and require confirmation in future studies. To explore potential sources of heterogeneity, subgroup analyses were prespecified with age (<18 vs. ≥18 years) and training-volume matching (matched vs. unmatched) as primary stratifying variables, and testing order and dominant-leg criteria as exploratory variables, contingent on at least 3 studies per subgroup. If any subgroup contained fewer than three studies, precluding meaningful statistical inference, we intended to refrain from meta-analytic pooling for that subgroup and instead provide descriptive analyses, in accordance with the recommendations of the Cochrane Handbook for Systematic Reviews of Interventions. For subgroup analyses, the I² statistic was recalculated within each subgroup, and the P value for subgroup difference was reported to evaluate effect modification.

Publication bias assessment: Given that the number of included studies for most outcomes was anticipated to be fewer than 10, the statistical power of the extended Egger’s regression test is substantially limited. Therefore, Egger’s test was not routinely applied as the primary method for detecting publication bias. Instead, publication bias was primarily assessed via visual inspection of funnel plots, which were interpreted qualitatively with caution due to the limited number of studies. For any specific outcome that included 10 or more eligible studies, Egger’s test was additionally performed as a complementary analysis, with the detailed results presented in the Supplementary Materials. When funnel plot asymmetry was observed, trim-and-fill analysis was not performed because the small number of studies (e.g., <10) precludes the generation of reliable adjusted effect estimates (Shi and Lin, 2019). Instead, we conducted sensitivity analyses using different correlation coefficients (r=0.5 and 0.8) for change-score variance calculations, as described above, to assess the robustness of our pooled results against potential bias, and by excluding studies rated as “high risk” or “some concerns” on the COD-specific items of the Risk of Bias assessment (e.g., non-randomized testing order), to test whether these methodological flaws influence the overall effect estimates for COD-L and COD-R.

For outcomes with critically small evidence bases (e.g., Number of studies=3, Number of participants<60), that underwent quantitative pooling, We therefore prespecified that: (1) narrative synthesis would take priority over pooled estimates for interpretation; (2) prediction intervals and leave-one-out sensitivity analyses would be computed to quantify uncertainty; and (3) all inferences would be explicitly designated as low-certainty exploratory evidence.

## Results

### Study selection

**Figure 1:**
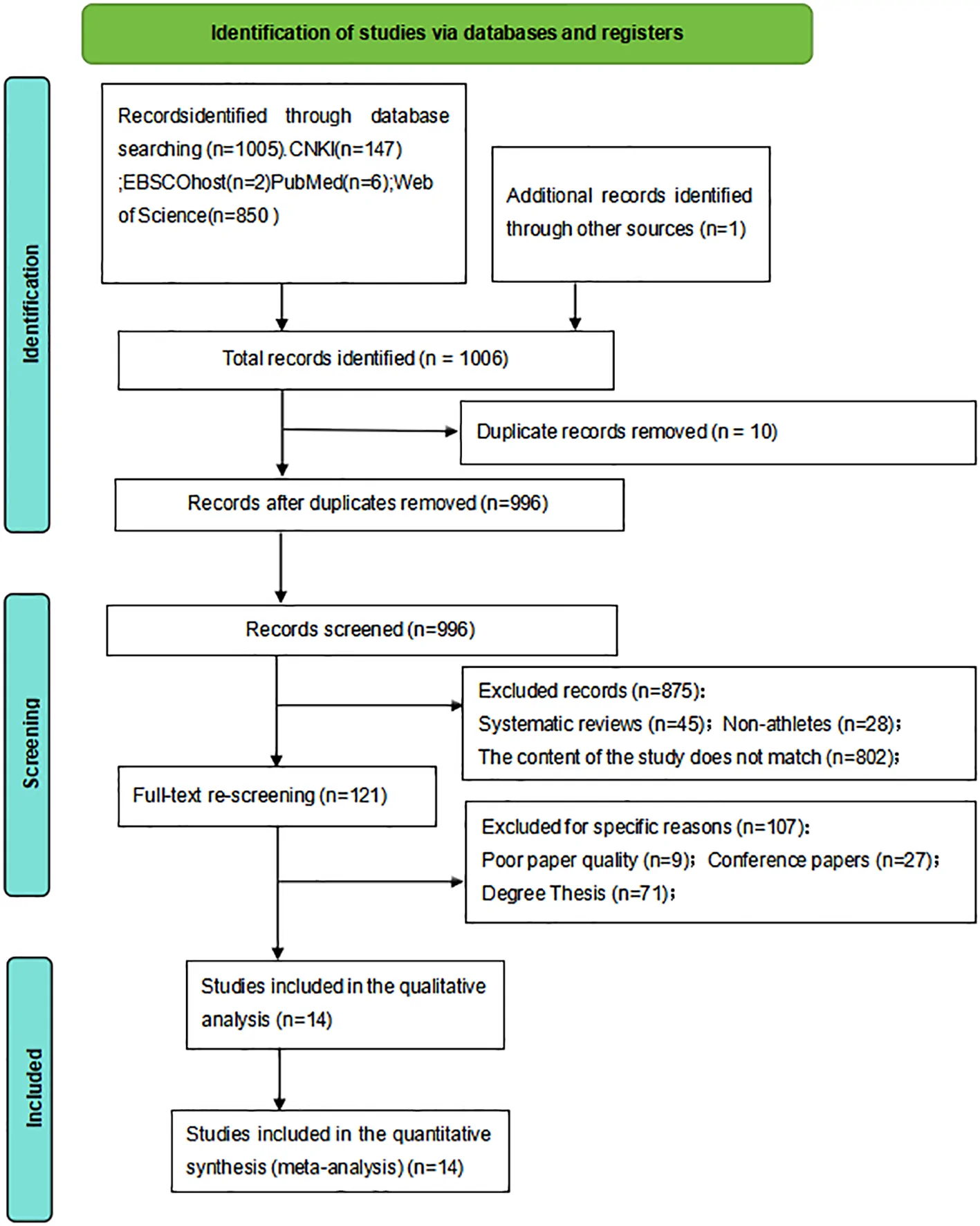
PRISMA flow diagram.

**Figure 1** illustrates the study selection process, which was conducted in accordance with the PRISMA 2020 guidelines. A total of 1,005 records were identified from four electronic databases, and one additional record was retrieved from Google Scholar and other sources, resulting in 1,006 records in total.

The screening process was performed in four stages. First, duplicate records identified during the search were removed (n=10), leaving 996 records for title and abstract screening. At this stage, 875 records were excluded because they did not involve athletes, were systematic reviews, or were otherwise irrelevant to the research topic, resulting in 121 reports proceeding to full-text assessment. Two reviewers (ZYG and SM) independently assessed the full texts of the remaining reports. To determine whether an intervention met the criterion of being “planned and structured,” the reviewers evaluated whether the training protocol included clear descriptions of training frequency, intensity, volume, duration, and progression. During the full-text assessment, 107 reports were excluded for the following reasons: 71 were unpublished dissertations (not peer-reviewed); 27 were conference papers lacking full-text data or sufficient methodological detail for effect size extraction; and 9 were excluded due to insufficient methodological quality (e.g., lack of a control group, inadequate description of the intervention, or high risk of bias). Any disagreements between the two reviewers were resolved through consultation with a third reviewer (CD) until consensus was reached. Ultimately, 14 studies met the inclusion criteria and were included in the final analysis. The numbers at each stage of the screening process were internally consistent (see **Figure 1**).

### Study quality assessment

The PEDro scale was used to assess the methodological quality of the included studies (see **Table 2**). The results showed that the scores of the 14 included studies ranged from 4 to 5, indicating moderate methodological quality (Maher et al., 2003).

**Table 2:** Physiotherapy evidence database (PEDro) scale ratings.

Study name	N1	N2	N3	N4	N5	N6	N7	N8	N9	N10	N11	Total^*^	Study quality
Chahal et al.(2026)	1	1	0	1	0	0	0	0	1	1	1	5	M
Li et al. (2025)	1	1	0	1	0	0	0	0	1	1	1	5	M
Zhang and Li (2026)	1	1	0	1	0	0	0	0	1	1	1	5	M
Núñez et al. (2018)	1	1	0	1	0	0	0	0	1	1	1	5	M
Stern et al. (2020)	1	1	0	1	0	0	0	0	1	1	1	5	M
Drouzas et al. (2020)	1	1	0	1	0	0	0	0	1	1	1	5	M
Belegišanin et al. (2025)	1	1	0	0	0	0	0	0	1	1	1	4	M
Fisher and Wallin (2014)	1	1	1	1	0	0	0	0	0	1	1	5	M
Gonzalo-Skok et al. (2017)	1	1	0	1	0	0	0	0	1	1	1	5	M
Speirs et al (2016)	1	1	0	0	0	0	0	0	1	1	1	4	M
Appleby et al. (2020)	1	1	0	0	0	0	0	0	1	1	1	4	M
Li et al. (2023)	1	1	0	0	0	0	0	0	1	1	1	4	M
Zhao et al. (2023)	1	1	0	0	0	0	1	0	1	1	1	5	M
Neves et al. (2025)	1	1	0	0	0	0	1	0	1	1	1	5	M

A detailed explanation for each PEDro scale item can be accessed at https://www.pedro.org.au/english/downloads/pedro-scale. *From a possible maximal punctuation of 10.M: stands for Moderate, H: stands for High.

### Risk of bias assessment results

**Figure 2:**
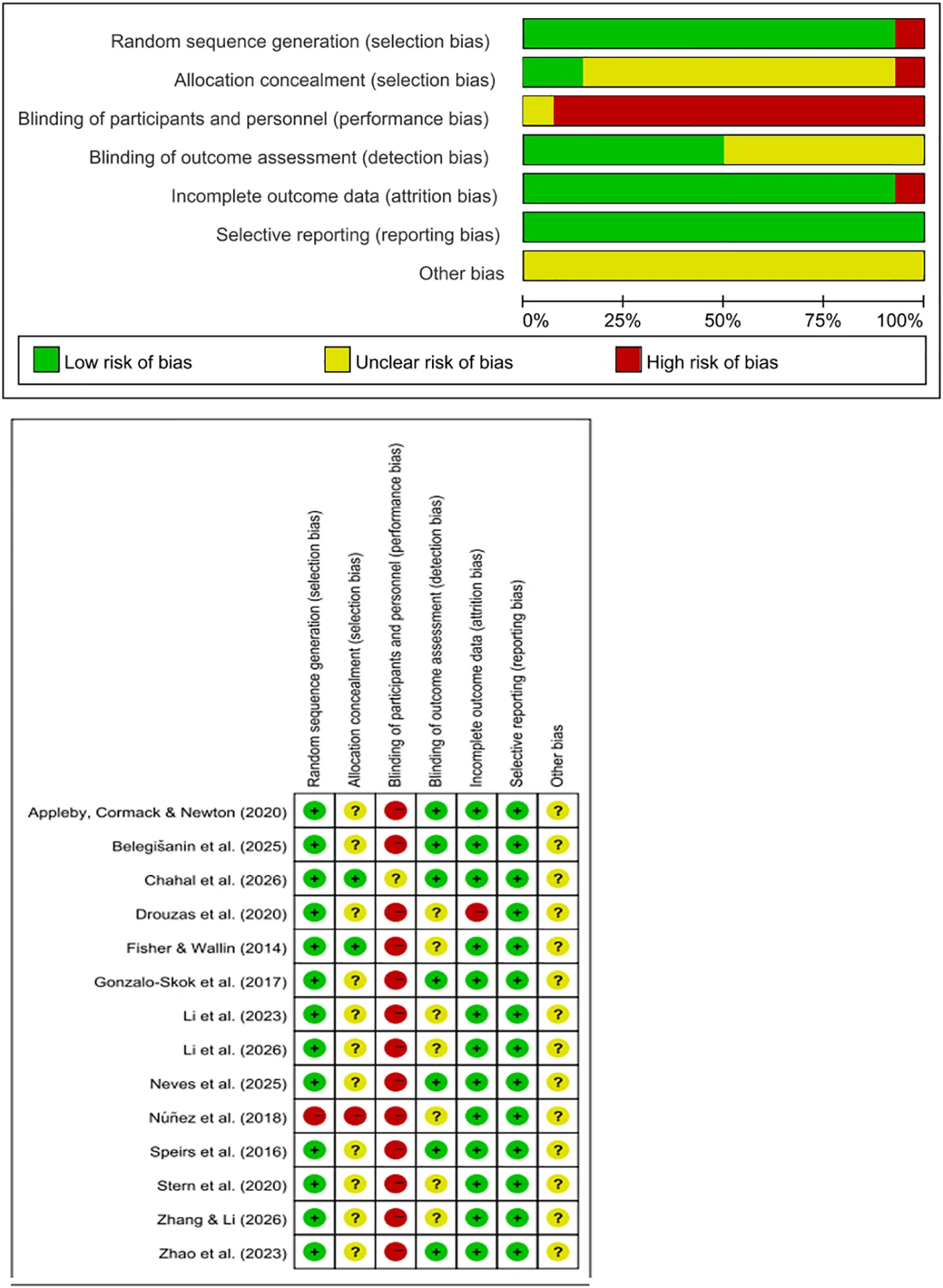
Risk of bias assessment using the original Cochrane risk of bias tool (Version 1.0). Green, yellow, and red indicate low, unclear, and high risk, respectively.

Two reviewers (CD and SM) independently assessed the risk of bias for the 14 included studies using the Cochrane Risk of Bias tool (RevMan 5.4.1). For each of the seven domains, studies were classified as having low risk, unclear risk, or high risk, with the overall risk-of-bias judgment subsequently determined based on domain-level ratings and study-specific characteristics (**Figure 2**). The assessment revealed a heterogeneous methodological quality profile. Specifically, while the majority of studies demonstrated low risk in random sequence generation and incomplete outcome data, performance bias was predominantly rated as high risk due to the inherent impossibility of blinding participants and personnel in exercise-based interventions. Additionally, a substantial proportion of studies were judged as unclear risk in allocation concealment and other bias domains, primarily owing to insufficient reporting in the original publications. Consequently, although certain domains were methodologically robust, the overall risk of bias across the included evidence base was considered moderate to high. Therefore, the pooled findings should be interpreted with caution, particularly regarding potential expectation effects and detection bias, which cannot be ruled out even when double-blinding is practically unattainable.

### Study characteristics

**Table 3** presents the characteristics of participants and intervention protocols of the randomized controlled trials included in this review. The publication years of the included studies ranged from 2014 to 2026. Among the included studies, one study used a four-group design: a unilateral plyometric plus sprint training group, a bilateral plyometric plus sprint training group, a sprint-only group, and an untrained control group (Zhang and Li, 2026), four studies used a three-arm design (Drouzas et al., 2020; Appleby et al., 2020; Zhao et al., 2023; Neves et al., 2025), and nine studies adopted a two-arm design (Chahal et al., 2026; Li et al., 2025; Núñez et al., 2018; Stern et al., 2020; Belegišanin et al., 2025; Fisher and Wallin, 2014; Gonzalo-Skok et al., 2017; Speirs et al., 2016; Li et al., 2023). Geographically, three studies were conducted in China (Li et al., 2025; Zhang and Li, 2026; Li et al., 2023), four in the United Kingdom (Stern et al., 2020; Fisher and Wallin, 2014; Speirs et al., 2016; Zhao et al., 2023), two in Spain (Núñez et al., 2018; Gonzalo-Skok et al., 2017), and one each in Germany (Chahal et al., 2026), Greece (Drouzas et al., 2020), Serbia (Belegišanin et al., 2025), Australia (Appleby et al., 2020), and Brazil (Neves et al., 2025). In terms of sporting population, five studies recruited football players (Chahal et al., 2026; Stern et al., 2020; Drouzas et al., 2020; Li et al., 2023; Neves et al., 2025), four studies involved basketball players (Li et al., 2025; Zhang and Li, 2026; Belegišanin et al., 2025; Gonzalo-Skok et al., 2017), four studies included rugby players (Fisher and Wallin, 2014; Speirs et al., 2016; Appleby et al., 2020; Zhao et al., 2023), and one study recruited team sport athletes (Núñez et al., 2018). A total of 415 male athletes were enrolled across all studies, with ages ranging from 9 to 27 years. With respect to competitive level, seven studies recruited collegiate athletes, two recruited academy-level athletes, one recruited national-level athletes, and four recruited regional-level athletes. This version accurately reflects the verified data (maximum training experience = 5 years, two studies with unreported training experience, and 10 studies with unreported session duration) and uses standard academic phrasing. If you prefer a slightly more formal alternative, you could replace “Notably” with “It is noteworthy that” or use “of note,” but the above is already journal-ready. In addition, the methods used to equate training volume between unilateral and bilateral training groups were not uniform across the included studies, and some studies did not clearly report their specific matching procedures in the methods section. Due to the limited number of included studies, we were unable to perform quantitative subgroup analyses or meta-regression to examine this methodological factor. Therefore, the possibility that the pooled effect estimates may be confounded by differences in training dose cannot be excluded, and the findings should be interpreted with caution.

**Table 3:** Characteristics of participants and interventions in the included studies.

Study	Country	Intervention	N	Sex	Age	Level/ Experience	Sport	Comparison	Outcome (s)
Chahal et al. (2026)	Germany	Freq: 2 times/ week Time: NR Length: 4weeks	42	M	EG:20.90 ± 2.57 CG:21.76 ± 2.30	District level > 6 months	Football	EG : Unilateral cycling CG : Bilateral cycling	30m↑
Li et al. (2025)	China	Freq: 2 times/ week Time: 40-45mins Length: 8 weeks	20	M	EG:20.6 ± 1.5 CG:20.4 ± 1.4	Collegiate >5 yrs	Basketball	EG : Unilateral plyometric training CG : Bilateral plyometric training	10m→ 505↑ COD-L↑ COD-R→
Zhang and Li (2026)	China	Freq: 2 times/ week Time: 45-55mins Length: 8 weeks	52	M	EG3:15.36 ± 1.43 CG:16.27 ± 1.10 EG1:15.90 ± 1.64 EG2:15.45 ± 1.46	Collegiate >3 yrs	Basketball	CG : Bilateral plyometric training + linear sprint training EG1:Linear sprint training only group EG2:Untrained control group EG3:Unilateral plyometric training + linear sprint training	10m↑ 20m↑ 505↑
Núñez et al. (2018)	Spain	Freq: 2 times/ week Time: NR Length: 6 weeks	27	M	EG1:22.8 ± 2.9 EG2:22.6 ± 2.7	Collegiate NR	Team sports	EG : Unilateral lunge squat CG : Bilateral lunge squat	T-Test→ COD↑
Stern et al. (2020)	UK	Freq: 2 times/ week Time: NR Length: 6 weeks	23	M	17.6 ± 1.2	District level> 2 yrs	Football	EG : Rear-foot elevated Bulgarian split squat, single-leg vertical jump, single-leg drop jump, single-leg standing long jump CG : Back squat, double-leg vertical jump, double-leg drop jump, double-leg standing long jump	10M↑ 30M→ COD-L→ COD-R↑
Drouzas et al. (2020)	Greece	Freq: 2 times/ week Time:10 mins Length: 10weeks	68	M	EG1:9.9 ± 1.8 EG2:10.0 ± 0.5 CG:10.2 ± 1.6	District level >3 yrs	Football	EG1:Unilateral plyometric training EG2:Bilateral plyometric training CG : Traditional training	5m↑ 10m→ 20m→ T-Test↑
Belegišanin et al. (2025)	Serbia	Freq: 4 times/ week Time: 90min Length: 6weeks	22	M	EG:15.5± 0.5 CG:15.2± 0.4	District level >4 yrs	Basketball	EG : Unilateral flywheel strength training CG : Bilateral flywheel strength training	5m↑ 20m↑ 505↑
Fisher and Wallin (2014)	UK	Freq: 2 times/ week Time: NR Length: 6weeks	15	M	EG:19.8 ± 1.49 CG:20.14 ± 1.77	collegiate level >2 yrs	Rugby union	EG : Unilateral resistance training CG : Bilateral resistance training	10m↓ T-Test↑
Gonzalo-Skok et al. (2017)	Spain	Freq: 2 times/ week Time: NR Length: 6weeks	22	M	EG:16.8 ± 1.7 CG:16.7 ± 1.7	National level Athlete >2 yrs	Basketball	EG : Unilateral resistance training CG : Bilateral resistance training	5 m→ 15 m→ 25m→ COD-R → COD-L ↑
Speirs et al. (2016)	UK	Freq: 2 times/ week Time: NR Length:5weeks	18	M	EG:18.1 ± 0.5 CG:18.1 ± 0.5	academy level>1 yrs	Rugby union	EG : Unilateral training CG : Bilateral training	10m↑; 40m↑;
Appleby et al. (2020)	Australia	Freq: 2 times/ week Time: NR Length: 12weeks	33	M	EG1:23.1 ± 4.1 EG2:21.8 ± 3.3 CG:24.6 ± 5.3	academy level >1 yrs	Rugby union	EG1:Unilateral resistance training EG2:Bilateral resistance training CG : Conventional training	5m 20m↑ COD→
Li et al. (2023)	China	Freq: 3 times/ week Time: NR Length: 6weeks	20	M	EG:17.0 ± 0. 33 CG:16.7 ± 0. 66	Collegiate > 2 yrs	Football	EG : Unilateral strength training CG : Bilateral strength training	30m↑ 505↑
Zhao et al. (2023)	UK	Freq: 2times/ week Time: NR Length: 5weeks	19	M	EG1:15.3 ± 0.3 EG2:15.2 ± 0.5 CG:15.3 ± 0.4	Collegiate >NR	Rugby union	EG1:Unilateral strength training EG2:Bilateral strength training CG : Conventional training	30m→
Neves et al. (2025)	Brazil	Freq: 2times/ week Time: NR Length: 4weeks	34	M	EG1:16.5 ± 0.9 EG2:17.0 ± 1.1 CG:16.9 ± 1.1	Collegiate > 2 yrs	Football	EG1:Unilateral isometric training EG2:Bilateral isometric training CG : Conventional training	10m→ 20m→ T-Test→

F, female; M, male; yrs, years; Freq, frequency; NR, not reported; EG, experimental group; CG, control group; ↑, significant within-group improvement.

→, no significant within-group improvement.

### Meta-analysis results

Among the included studies, fourteen studies assessed athletes’ linear sprint speed, with testing protocols comprising 5 m, 10 m, 20 m, and 30 m sprint performances. In addition, a range of change-of-direction speed metrics were employed across the included trials, including the T-test, 505 test, and COD-L and COD-R tests. As the majority of outcomes included fewer than 10 studies, formal publication bias assessments (e.g., funnel plots) were not performed, consistent with our prespecified analytical approach.

### Meta-analysis of unilateral versus bilateral training on linear sprint performance

**Figure 3:**
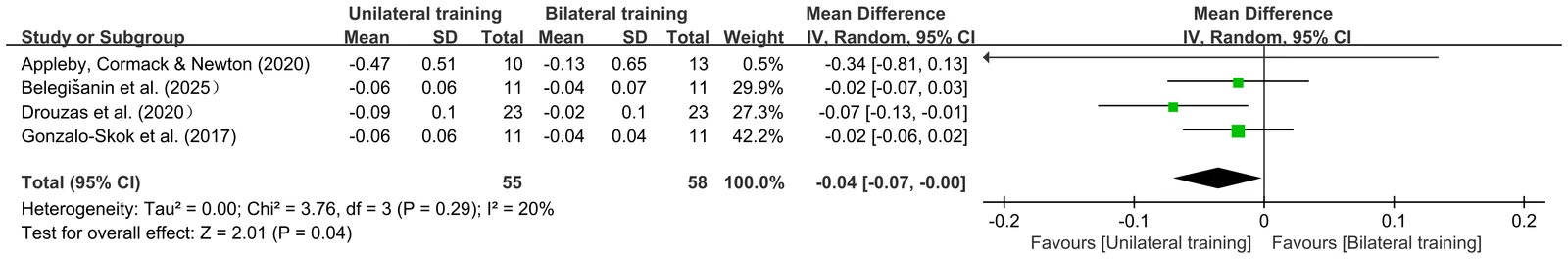
Forest plot of the effects of unilateral versus bilateral training on athletes’ 5 m sprint performance. Values represent mean differences (MD) in seconds with 95% confidence intervals (CI).

Among the included studies, four provided pre- and post-training data on the effects of unilateral versus bilateral training on 5 m sprint performance in athletes, involving four experimental groups and four control groups (113 athlete participants in total). As shown in **Figure 3**, a random-effects model was used for the pooled analysis. The results yielded a pooled mean difference of −0.04 (95% CI: −0.07 to −0.00) in favor of unilateral training, with a Z-statistic of 2.01 (*p* = 0.04), indicating a statistically significant difference between groups. The heterogeneity analysis showed low statistical heterogeneity across the four studies (*p* = 0.29, *I²* = 20%), though this estimate should be interpreted with prudence due to the limited number of pooled trials.

**Figure 4:**
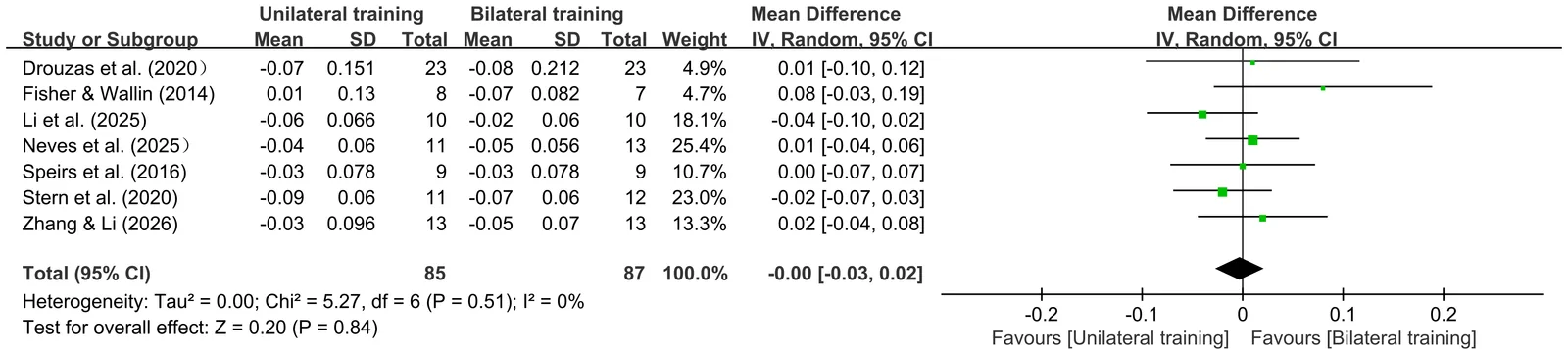
Forest plot of the effects of unilateral versus bilateral training on athletes’ 10 m sprint performance. Values represent mean differences (MD) in seconds with 95% confidence intervals (CI).

Among the included studies, seven provided pre- and post-training data on the effects of unilateral versus bilateral training on 10 m sprint performance in athletes, involving seven experimental groups and seven control groups (172 athlete participants in total). As shown in **Figure 4**, a random-effects model was used for the pooled analysis. The results yielded a pooled mean difference of 0.00 (95% CI: −0.03 to 0.02), with a Z-statistic of 0.20 (*p* = 0.84), indicating no statistically significant between-group difference. In addition, low heterogeneity was observed across studies (*p* = 0.51, *I²* = 0%), suggesting a relatively high consistency in the direction of effects among the included studies.

**Figure 5:**
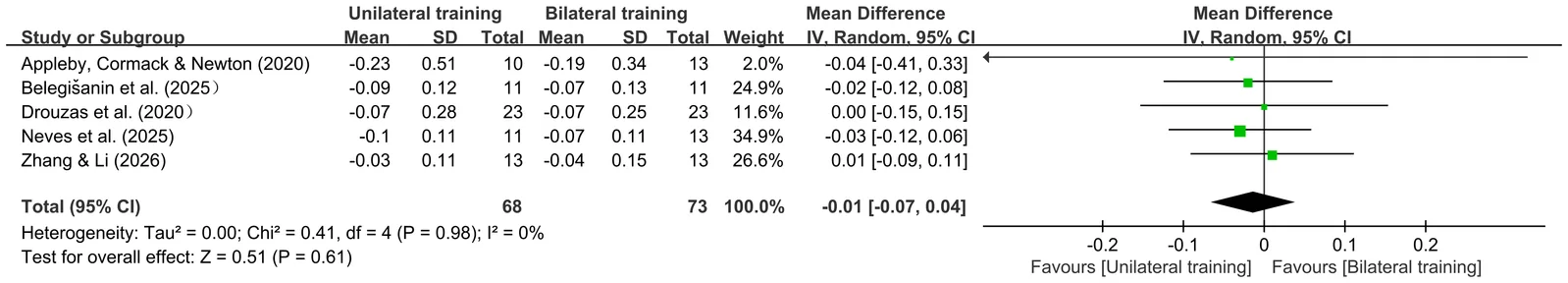
Forest plot of the effects of unilateral versus bilateral training on athletes’ 20 m sprint performance. Values represent mean differences (MD) in seconds with 95% confidence intervals (CI).

Among the included studies, five provided pre- and post-training data on the effects of unilateral versus bilateral training on 20 m sprint performance in athletes, involving five experimental groups and five control groups (141 athlete participants in total). As shown in **Figure 5**, a random-effects model was applied for the pooled analysis. The results yielded a pooled mean difference of −0.01 (95% CI: −0.07 to 0.04), with a Z-statistic of 0.51 (*p* = 0.61), indicating no statistically significant difference between the two training modalities. In addition, heterogeneity tests revealed negligible variability across studies (Tau² = 0.00, Chi² = 0.41, *df* = 4, *p* = 0.98, *I²* = 0%), suggesting a relatively high consistency in the direction of effects among the included studies.

**Figure 6:**
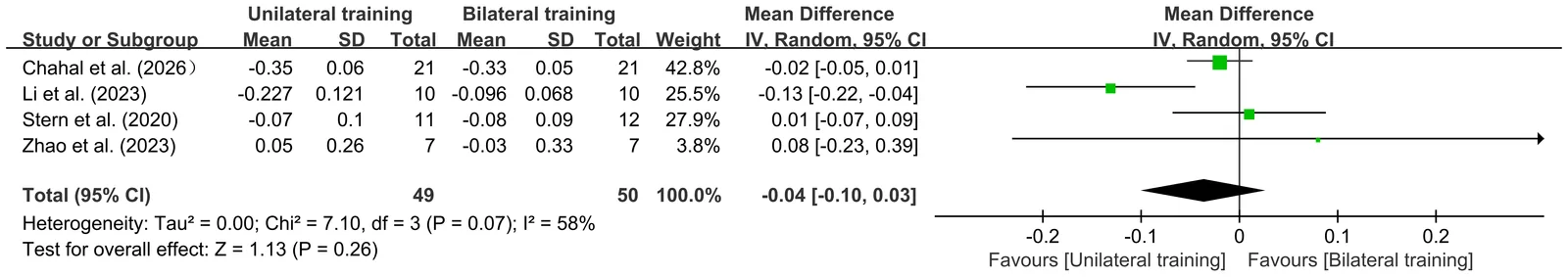
Forest plot of the effects of unilateral versus bilateral training on athletes’ 30 m sprint performance. Values represent mean differences (MD) in seconds with 95% confidence intervals (CI).

Among the included studies, four provided pre- and post-training data on the effects of unilateral versus bilateral training on 30 m sprint performance, involving a total of 99 athletes (49 in the unilateral group and 50 in the bilateral group). As shown in **Figure 6**, a random-effects model was used for the pooled analysis due to the presence of moderate-to-substantial heterogeneity (*I²* = 58%). The results yielded a pooled mean difference of −0.04 (95% CI: −0.10 to 0.03), with a Z-statistic of 1.13 (*p* = 0.26), indicating no statistically significant difference between groups. Notably, when the analysis was repeated using a fixed-effect model, the confidence interval narrowed and showed a nominally significant difference favoring unilateral training (p < 0.05); however, this significance was not robust, as it disappeared under the more conservative random-effects model. Furthermore, leave-one-out sensitivity analysis revealed that the pooled estimate was substantially influenced by the study of Li et al. (2023), although the overall effect remained non-significant after its exclusion (data not shown). Given the moderate heterogeneity (*p* = 0.07, *I²* = 58%) and inconsistent effect directions across studies, these findings should be interpreted with caution, and further investigation is warranted.

### Meta-analysis of unilateral versus bilateral training on change-of-direction performance

**Figure 7:**
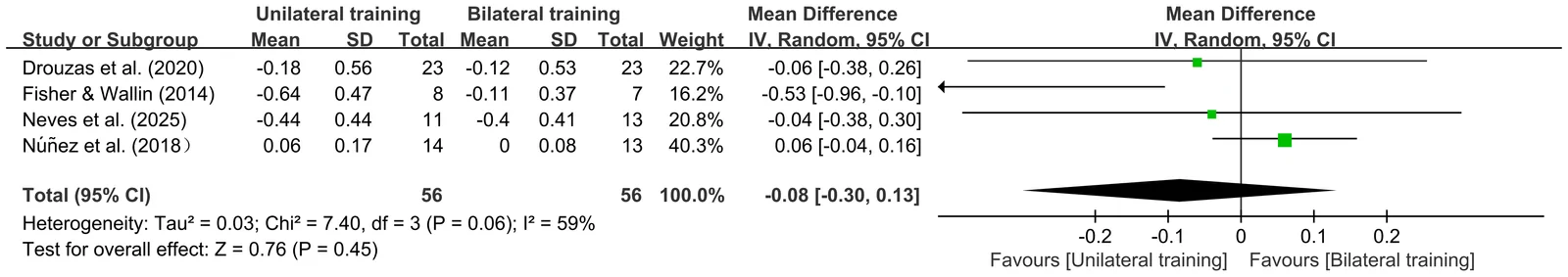
Forest plot of the effects of unilateral versus bilateral training on athletes’ T-test agility performance. Values represent mean differences (MD) in seconds with 95% confidence intervals (CI).

Among the included studies, four provided pre-and post-intervention data on the effects of unilateral versus bilateral training on T-agility test performance in athletes, involving four experimental groups and four control groups with a total of 112 participants. As shown in **Figure 7**, a random-effects model was used for the pooled analysis, which revealed no statistically significant difference between unilateral and bilateral training, with a pooled mean difference of −0.08 (95% CI: −0.30 to 0.13) and a test statistic of Z = 0.76 (*p* = 0.45).

Regarding heterogeneity, moderate heterogeneity was observed across studies (χ²=7.40, *df* = 3, *p* = 0.06; *I²* = 59%), suggesting a certain degree of true variation in effect sizes among the studies. Given that the I² value fell within the moderate range (25–75%) prespecified in our analytical framework, the pooled estimate was considered interpretable while acknowledging the moderate between-study variability. At the individual study level, Fisher and Wallin (2014) reported that unilateral training was significantly superior to bilateral training (MD=−0.53, 95% CI: −0.96 to −0.10), whereas Drouzas et al. (2020) and Neves et al. (2025) found no significant differences between groups (with confidence intervals crossing zero). Núñez et al. (2018) had a point estimate favoring bilateral training, but its confidence interval also included zero (MD = 0.06, 95% CI: −0.04 to 0.16). In summary, given the non-significant overall effect and the inconsistent direction of effects across studies, these findings should be interpreted with caution.

**Figure 8:**
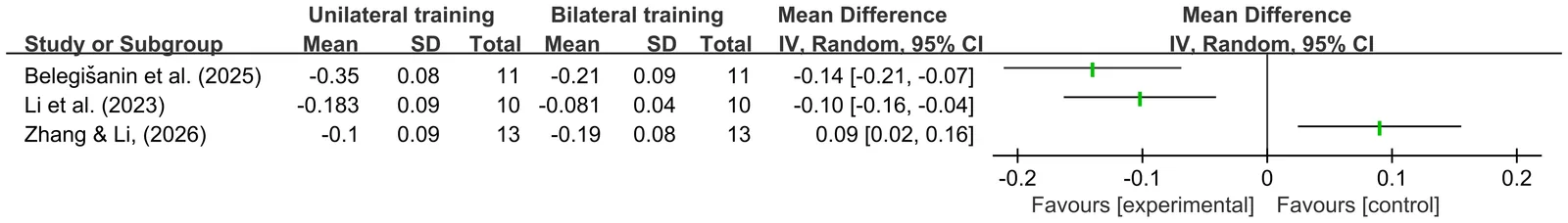
Forest plot of individual study results for the effect of unilateral versus bilateral training on 505 performance. Due to substantial heterogeneity (I² = 92%), quantitative pooling was not performed; therefore, no summary estimate is presented.

Among the included studies, three provided pre- and post-intervention data on the effects of unilateral versus bilateral training on 505 performance, involving three experimental groups and three control groups with a total of 68 athlete participants (As shown in **Figure 8**). In accordance with our prespecified analytical framework (see Statistical analysis), quantitative pooling (meta-analysis) was not performed for this outcome owing to the extremely high heterogeneity (*I²* = 92%). Instead, the findings from individual studies are summarized descriptively. At the individual study level, Belegisinin et al. (2025) reported that unilateral training was significantly superior to bilateral training (MD = −0.14, 95% CI: −0.21 to −0.07), and Li et al. (2023) similarly found a significant advantage for unilateral training (MD = −0.10, 95% CI: −0.16 to −0.04). In contrast, Zhang and Li (2026) reported a significant advantage for bilateral training (MD = 0.09, 95% CI: 0.02 to 0.16). The direction of effects was inconsistent across studies, with two studies favoring unilateral training and one favoring bilateral training. Given the limited number of studies (n = 3) and the substantial between-study heterogeneity (*I²* = 92%), no definitive conclusion can be drawn regarding the effect of unilateral versus bilateral training on 505 performance. These findings should be interpreted as low-certainty exploratory evidence, and future studies with standardized testing protocols and larger sample sizes are warranted to clarify this outcome. 

**Figure 9:**
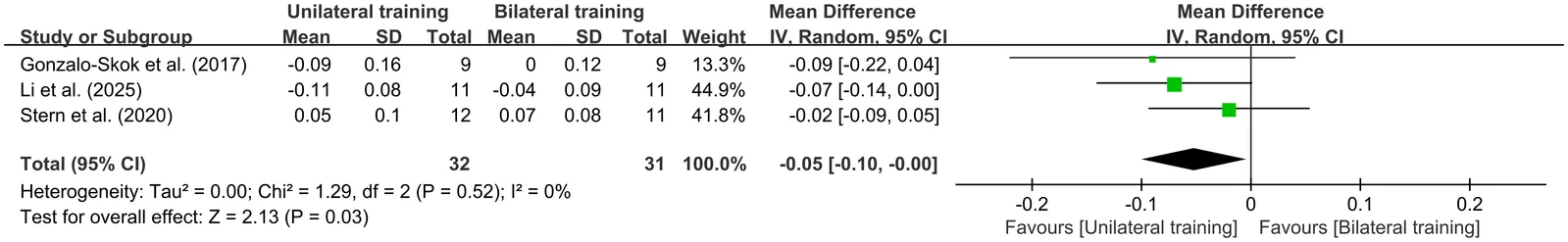
Forest plot of the effects of unilateral versus bilateral training on athletes’ COD-L performance. Values represent mean differences (MD) in seconds with 95% confidence intervals (CI).

Among the included studies, three provided pre- and post-intervention data on the effects of unilateral versus bilateral training on COD-L (left-side change-of-direction) performance, involving three experimental groups and three control groups with a total of 63 athlete participants. As shown in **Figure 9**, a random-effects model was used for the pooled analysis. The results yielded a pooled mean difference of −0.05 (95% CI: −0.10 to −0.00), with a test statistic of Z = 2.13 (*p* = 0.03), indicating a borderline statistically significant advantage for unilateral training in this exploratory analysis; however, the proximity of the upper confidence limit to the null value warrants caution. The I² statistic showed no heterogeneity (0%) across the three studies (*p* = 0.52), suggesting low heterogeneity, but this finding should be interpreted cautiously given the minimal sample size and the limited number of pooled studies.

**Figure 10:**
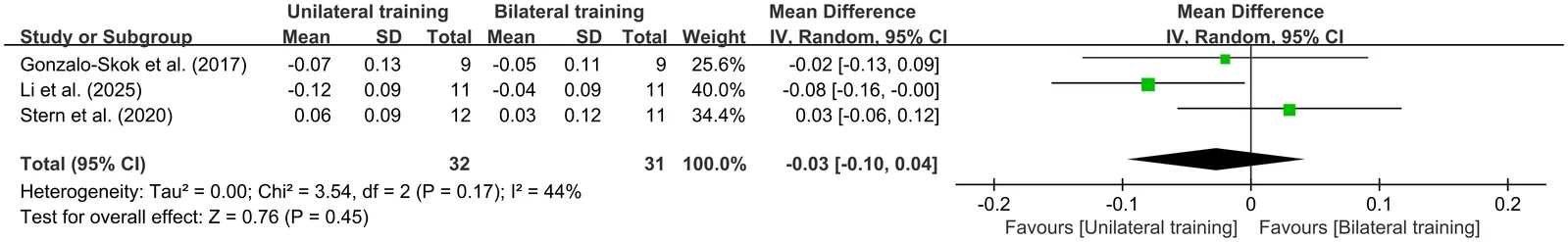
Forest plot of the effects of unilateral versus bilateral training on athletes’ COD-R performance. Values represent mean differences (MD) in seconds with 95% confidence intervals (CI).

Among the included studies, three provided pre- and post-training data on the effects of unilateral versus bilateral training on COD-R (right-side change-of-direction) performance in athletes, involving three experimental groups and three control groups (63 athlete participants in total). As shown in **Figure 10**, a random-effects model was used for the pooled analysis. The results yielded a pooled mean difference of −0.03 (95% CI: −0.10 to 0.04), Z = 0.76 (*p* = 0.45), indicating no statistically significant difference between groups. In addition, moderate heterogeneity was observed across studies (*p* = 0.17, *I²* = 44%), which falls within the moderate range (25–75%) prespecified in our analytical framework, justifying the use of the random-effects model.

### Additional analysis

**Figure 11:**
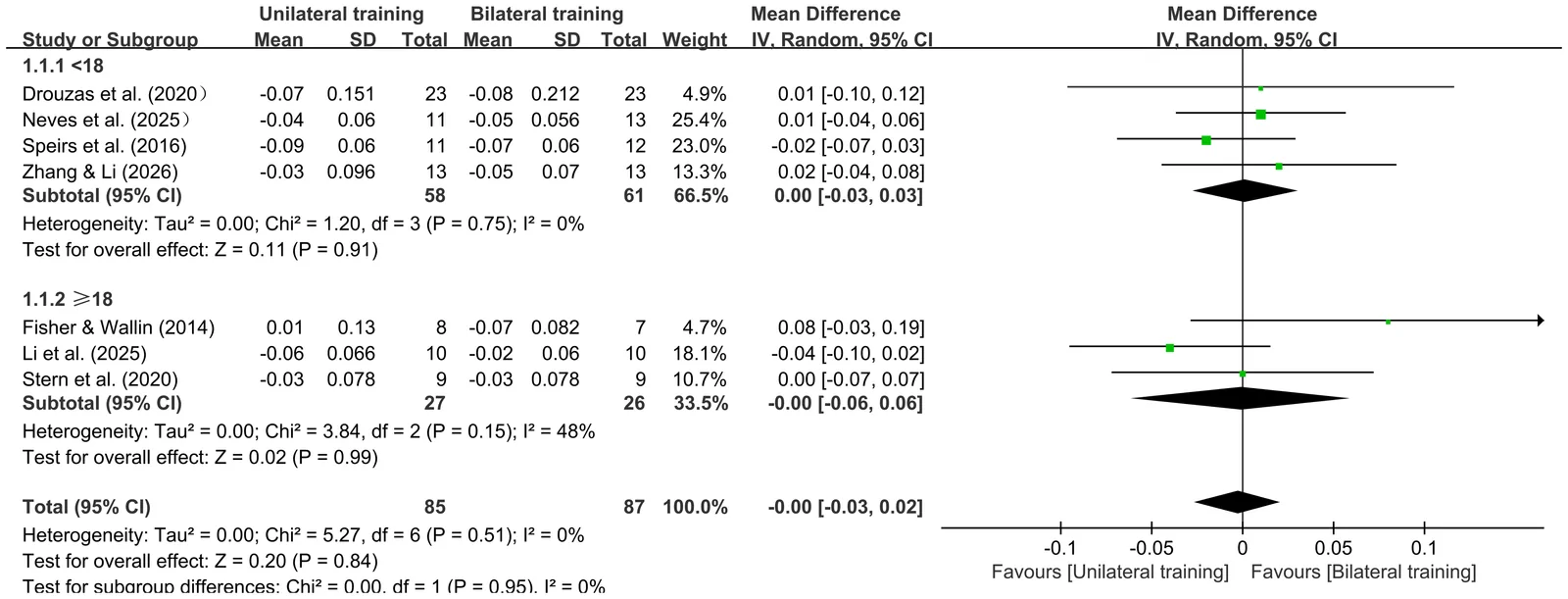
Subgroup forest plot of the effects of unilateral versus bilateral training on 10 m performance in athletes, stratified by age (<18 years vs. ≥18 years). Values represent mean differences (MD) in seconds with 95% confidence intervals (CI).

We performed a subgroup analysis based on participants’ age to examine its potential moderating effect on 10 m sprint performance in athletes. The random-effects model showed that age (<18 years vs. ≥18 years) did not significantly moderate the training effects (*P* = 0.95). Both the adolescent subgroup (MD = 0.00, 95% CI: −0.03 to 0.03, *P* = 0.91) and the adult subgroup (MD = −0.00, 95% CI: −0.05 to 0.05, *P* = 0.99) showed no significant differences between unilateral and bilateral training. The overall pooled effect did not support the superiority of unilateral training over bilateral training (MD = 0.00, 95% CI: −0.03 to 0.02, *P* = 0.84; **Figure 11**).

### Adverse effects

Across all included studies, no adverse effects related to unilateral training interventions were reported, including pain, injuries, or other health-related issues among athletes.

## Discussion

This study aimed to investigate the effects of unilateral training compared with bilateral training on athletes’ linear sprint speed and change-of-direction (COD) ability. Based on predefined inclusion and exclusion criteria, 14 studies were ultimately included for analysis. The included studies were categorized and quantitatively synthesized according to the outcome measures reported. The results indicated that, in terms of linear sprint performance, unilateral training exhibited a significant effect on 5-m sprint performance compared with bilateral training; in terms of COD performance, unilateral training showed a significant effect on left-side COD (COD-L), whereas no significant effects were observed for other sprint distances (10 m, 20 m, and 30 m) or for right-side COD (COD-R). Overall, these findings suggest that unilateral training may confer phase-specific advantages over bilateral training for specific linear sprint and COD measures; however, given that no adjustment for multiple comparisons was made, these results should be considered exploratory.

### Effects of unilateral training on athletes’ linear sprint performance

Based on the meta-analytic results regarding athletes’ linear sprint performance, the benefits of unilateral training appear to be selective and phase-specific(i.e., acceleration phase rather than maximal velocity phase), indicating a clear boundary of applicability. Specifically, the findings show that unilateral training produced a notable effect on 5 m sprint performance, whereas no notable effects were observed for 10 m, 20 m, or 30 m sprint performance under the random-effects model. For the 30 m sprint, the moderate heterogeneity (*I²* = 58%) and sensitivity analyses support the null finding. These results are consistent with previous meta-analytic evidence comparing unilateral and bilateral training on sprint performance (Moran et al., 2021), which also reported limited advantages of unilateral training in enhancing linear sprint speed. Notably, while the I² values indicated low to moderate heterogeneity (<75%) for the significant outcomes, this does not confirm the efficacy of unilateral training per se, but rather suggests that the pooled estimates were relatively stable across studies for the 5 m sprint outcome.

These findings suggest that unilateral training may serve as a complementary, rather than a primary, training modality for improving athletes’ linear sprint performance. However, given that among the included outcome measures related to linear sprint performance, only the 5 m sprint showed a significant effect, caution is warranted regarding the practical application of unilateral training as a training modality. This provides a clearer practical understanding of the relationship between training methods and linear sprint performance.

From a theoretical perspective, training adaptations are widely understood to follow the principle of specificity, which states that training outcomes are determined by the degree of correspondence between training stimuli (e.g., intensity, force direction, and contraction type) and performance goals (Kanehisa and Miyashita, 1983). Furthermore, prior research has demonstrated that sprint performance is not a single construct but rather consists of multiple phases with distinct mechanical demands and neuromuscular control characteristics (Mero et al., 1992; Ross et al., 2001).

In line with these theoretical foundations, the present findings further highlight that the effectiveness of training interventions depends on whether they target the key determinants of specific speed phases. In particular, unilateral training was found to be effective for improving 5 m sprint performance—a phase primarily reflecting starting acceleration and explosive power. but showed no significant effects on longer sprints (10–30 m), which involve greater demands on maximum velocity maintenance and technical coordination. This supports the concept of phase-specific speed development and reinforces the applicability of the training specificity principle in sprint training, while also highlighting differentiated performance characteristics across speed phases. Notably, the included participants spanned a wide age range (9–27 years). In the 9–17 years subgroup, neuromuscular adaptations may be substantially influenced by pubertal maturation and training age rather than by chronological age alone—a factor that may partly explain the observed heterogeneity. From a practical perspective, these findings provide meaningful guidance for coaches in designing and optimizing linear speed training programs. Specifically, unilateral training should be applied in a differentiated manner according to training objectives and used as a supplementary rather than core method, thereby improving the scientific and precise structuring of speed training interventions.

Regarding intervention characteristics, we aimed to explore whether training protocols moderate the effects of unilateral training. However, due to the limited number of included studies, we were only able to conduct subgroup analyses based on participants’ age for the 10 m sprint outcome. Subgroup analyses showed that age (<18 years vs. ≥18 years) did not significantly moderate 10 m sprint performance (*P* = 0.95), and the comparison between unilateral and bilateral training did not reveal a significant difference (MD = 0.00, 95% CI: −0.03 to 0.02, *P* = 0.84). These findings suggest that the effects of unilateral training relative to bilateral training on linear sprint performance are relatively consistent across age groups and training modalities, although the limited sample sizes within subgroups constrain the statistical validity of the exploratory analyses. Future research should incorporate more primary studies and perform well-defined moderator variable analyses.

### Effects of unilateral training on athletes’ change-of-direction speed

Based on the meta-analytic results regarding athletes’ COD performance, the improvements conferred by unilateral training appear to be non-uniform and direction-dependent, with the magnitude of change being small in absolute terms (i.e., differences were measured in fractions of a second). For COD-R, the pooled estimate was null (MD=−0.03, 95% CI: −0.08 to 0.02, p=0.24), with the prediction interval (−0.17 to 0.07) crossing zero and leave-one-out analysis indicating that the result was driven primarily by Li et al. (2025). Specifically, among the included COD-related indicators (e.g., T-test, 505 test, and COD tests), only COD-L showed a notable advantage of unilateral training over bilateral training. However, it should be noted that the number of included studies reporting COD-L outcome measures is limited, and the observed direction-specific effect warrants further confirmation. These findings indicate that the influence of unilateral training on change-of-direction ability is specific to certain movement directions rather than universally applicable across all COD tasks.

This result is consistent with previous literature (Zhang et al., 2025), which also reported that the benefits of unilateral training on change-of-direction performance are asymmetrical. Compared with prior studies, the present meta-analysis specifically focused on the independent effects of unilateral training on athletic performance, minimizing potential confounding effects from mixed training modalities, thereby providing further evidence for the directional specificity of unilateral training effects on COD performance.

The meta-analysis results indicated that unilateral training was effective for COD-L but not for COD-R in athletes. This differential effect may be attributed to several factors. First, COD-L and COD-R involve different hip rotation angles, foot placement, and ground reaction force vectors, which may result in divergent responses to the same training stimulus (Dos’Santos et al., 2019). Second, given that the majority of participants were right-leg dominant, unilateral training may have produced more pronounced improvements on the dominant side, and the varying demands on the supporting leg between COD-L and COD-R could contribute to directional asymmetry (Dos’Santos et al., 2018). From a biomechanical perspective, during cutting maneuvers, the support leg serves as the primary force-producing limb. In COD-L tests, the dominant leg typically acts as the support leg, whereas COD-R predominantly relies on the non-dominant leg for this function. Consequently, unilateral training-induced neuromuscular adaptations disproportionately favor COD-L performance (Dos’Santos et al., 2020). However, most included studies did not explicitly report whether the dominant or non-dominant leg was used as the support leg during directional COD testing, which precludes a definitive biomechanical interpretation of the observed directional asymmetry. Therefore, caution should be exercised when applying these findings to practical settings.

From a theoretical perspective, change-of-direction performance is widely recognized as a complex motor skill that integrates deceleration, acceleration, and multi-directional coordination, relying heavily on force control and neuromuscular coordination (Chaouachi et al., 2012; Nimphius, 2017). The present findings further support the notion that COD ability is highly task-specific, with different directional tasks imposing distinct physiological and mechanical demands.

Regarding intervention characteristics, we aimed to examine whether training protocols moderated the effects of unilateral training on change-of-direction (COD) performance. However, owing to the limited number of included studies, robust subgroup analyses could not be conducted. Tentatively, descriptive comparisons suggested that longer interventions (≥8 weeks) and combined training (e.g., with plyometric or resistance exercises) tended to yield more favorable outcomes for COD-L (left-side change of direction). Nevertheless, the small study pool and insufficient statistical power for formal interaction testing limit the confirmatory nature of these observations.

### Additional discussion

The forest plot of the meta-analysis revealed extremely high heterogeneity for the 505 test (I²=92%), precluding quantitative pooling and diminishing the interpretability of this outcome. Several factors may contribute to this heterogeneity, including differences in training modalities that induce distinct neuromuscular adaptations, whether training volume was matched between unilateral and bilateral groups, and participant characteristics such as maturational status and training age (De Bosscher et al., 2009). However, our age-based subgroup analysis (<18 vs. ≥18 years) did not identify age as a significant moderator of 10 m sprint performance (P for subgroup difference=0.64). Methodological variations in COD testing protocols, such as testing order and criteria for determining the dominant leg, may also introduce additional variability. Given that only three studies reported 505 outcomes, we were unable to perform robust meta-regression or subgroup analyses to quantify the contribution of each factor; therefore, these proposed sources remain speculative. For outcomes with low-to-moderate heterogeneity (30 m sprint, I²=58%; T-test, I²=59%; COD-R, I² =44%), the pooled estimates are considered more reliable, although moderate heterogeneity for the 30 m sprint and T-test warrants caution in interpretation.

### Limitations

Several methodological limitations should be acknowledged when interpreting the present findings. First, although the included studies involved unilateral training combined with various training modalities across different sports, they did not comprehensively cover all training approaches related to athletes’ linear sprint and COD performance; therefore, the generalizability of the findings remains limited, particularly given that many studies did not clearly report directional definitions, support leg roles, or testing orders for COD-L and COD-R. In particular, the lack of explicit reporting on whether the dominant or non-dominant leg served as the support leg during directional COD testing precludes a definitive biomechanical interpretation of the observed directional asymmetry. Second, due to sample-related constraints, the characteristics of participants in the included studies could not fully represent all athletes in terms of age, sex, sport type, and competitive level. As a result, the applicability of the findings across diverse athletic populations is restricted. This is especially relevant given the wide age range of participants (9–27 years) and the potential influence of pubertal maturation on neuromuscular adaptations in the younger subgroup, which may have contributed to the observed heterogeneity. Third, the limited number of included studies prevented meaningful comparisons regarding whether differences in training duration and intensity influence the effects of unilateral training on linear sprint and COD performance, which warrants further investigation. Similarly, we were unable to perform robust meta-regression or formal interaction tests for most moderator variables (e.g., training volume, intervention duration, and sport type). Consequently, the external validity of the findings is constrained. Of note, the COD-L finding was derived from only three studies (63 athletes), rendering the 0% I² estimate unreliable; the 505 test exhibited extremely high heterogeneity (I² = 92%), precluding quantitative pooling; and multiple COD comparisons were examined without correction for multiplicity, raising the risk of Type I error. Fourth, only original studies published in Chinese and English were included, which may limit the representativeness of the evidence base. Fifth, according to the GRADE assessment, most included studies were rated as moderate-quality evidence, which may to some extent reduce the certainty of the reported effect estimates. Sixth, given that the number of included studies for most outcomes was fewer than 10, formal publication bias assessments (e.g., funnel plots and Egger’s regression tests) could not be reliably performed. Consequently, the potential presence of publication bias cannot be entirely ruled out, and the results should be interpreted with caution. Seventh, COD performance is a composite measure, typically expressed as total completion time, which makes it difficult to isolate contributions from different phases such as deceleration, acceleration, and turning. The reliance on single outcome metrics, particularly in the absence of biomechanical variables, limits deeper interpretation of the underlying mechanisms. Eighth, substantial methodological heterogeneity was observed across studies, particularly in intervention characteristics such as training intensity, duration, and combined training modalities, as well as in directional definitions and testing protocols for left/right COD assessments, which may limit the generalizability of the findings. Ninth, differences in sample size, experimental design, and measurement conditions across studies may have influenced the stability of effect size estimates. Moreover, although secondary data transformation was applied in the meta-analysis, the quality variability of the original studies may have affected the robustness of the synthesized results. Given these limitations, future studies should provide detailed and standardized reporting of training variables (e.g., intensity, volume, and frequency), participant characteristics (e.g., sport discipline and training age), and COD testing protocols, in order to facilitate explicit moderator analyses and meta-regressions. Furthermore, well-designed primary studies with larger sample sizes are warranted to directly compare unilateral and bilateral training regimens, and to determine whether the directional asymmetry in COD performance is consistently reproducible across diverse athletic populations.

## Conclusions

This systematic review and meta-analysis suggests that unilateral training may offer selective benefits for specific measures of linear sprint speed and change-of-direction performance in male athletes, including those from basketball, soccer, rugby, and other team sports. Specifically, unilateral training showed notable positive effects on 5 m sprint performance (Z = 2.27, p=0.02) and COD-L performance (Z = 2.13, p=0.03), whereas no significant effect was observed for COD-R, indicating a potential direction-specific effect. These findings suggest that incorporating unilateral training into periodized training programs may help optimize specific sprint and COD qualities in athletes. However, the current evidence base remains insufficient to inform specific training recommendations, and the primary contribution of this study is to identify key research gaps for further investigation.

### Practical application

The findings of this study provide practical guidance for athletes and coaches in improving linear sprint and COD performance, with the observation that the notable improvement was specific to COD-L, whereas COD-R showed no notable change, while also extending the understanding of the mechanisms underlying different training modalities, such as plyometric training, strength training, and isometric training, in athletic performance enhancement. In future training practice, unilateral training can be considered as a supplementary strategy to improve specific sprint and COD outcomes. One key advantage of these findings is that unilateral training can be incorporated into existing training systems without requiring major modifications to established training methods or structures. This makes it relatively easy for coaches to implement and allows athletes to acquire the training approach within a short period of time. However, given the variability in training protocols across the included studies, the optimal unilateral training prescription remains to be determined. Additionally, the methods used to equate training volume between unilateral and bilateral training groups varied across studies; therefore, the possibility that training intensity and frequency act as confounding variables cannot be excluded. Based on the characteristics of the included studies, coaches may consider implementing unilateral training as a potential supplementary intervention to improve 5 m sprint and COD-L performance, with the specific dose tailored to individual athlete needs and overall training plans.

## Data Availability

The original contributions presented in the study are included in the article/Supplementary Material. Further inquiries can be directed to the corresponding author.

## References

[B20] Appleby B. B. Cormack S. J. Newton R. U. (2019). Specificity and transfer of lower-body strength: influence of bilateral or unilateral lower-body resistance training. J. Strength Conditioning Res. 33, 318–326. doi: 10.1519/JSC.0000000000002923 30688873

[B40] Appleby B. B. Cormack S. J. Newton R. U. (2020). Unilateral and bilateral lower-body resistance training does not transfer equally to sprint and change of direction performance. J. Strength Conditioning Res. 34, 54–64. doi: 10.1519/JSC.0000000000003035 30844983

[B47] Belegišanin B. Andrić N. Jezdimirović Stojanović T. Ninkov A. Bajić G. Osmankač N. . (2025). A comparison of bilateral vs. unilateral flywheel strength training on physical performance in youth male basketball players. J. Funct. Morphology Kinesiology 10, 81. doi: 10.3390/jfmk10010081 40137333 PMC11943462

[B7] Bishop D. J. Beck B. Biddle S. J. H. Denay K. L. Ferri A. Gibala M. J. . (2024). Physical activity and exercise intensity terminology: a joint American college of sports medicine (ACSM) expert statement and exercise and sport science Australia (ESSA) consensus statement. J. Sci. Med. Sport 57, 2599–2613. doi: 10.1016/j.jsams.2024.11.004 41085254

[B8] Blazevich A. J. Jenkins D. G. (2002). Effect of the movement speed of resistance training exercises on sprint and strength performance in concurrently training elite junior sprinters. J. Sports Sci. 20, 981–990. doi: 10.1080/026404102321011742 12477008

[B43] Chahal J. Rizvi M. R. Sharma A. Nigam S. Sami W. (2026). Enhancing neuromuscular conditioning in football players through single-leg and double-leg cycling: a randomized controlled trial. Transl. Sports Med. 2026, 5535929. doi: 10.1155/tsm2/5535929 41768376 PMC12948725

[B58] Chaouachi A. Manzi V. Chaalali A. Wong D. P. Chamari K. Castagna C. (2012). Determinants analysis of change-of-direction ability in elite soccer players. J. Strength Conditioning Res. 26, 2667–2676. doi: 10.1519/JSC.0b013e318242f97a 22124358

[B60] De Bosscher V. De Knop P. van Bottenburg M. (2009). An analysis of homogeneity and heterogeneity of elite sports systems in six nations. Int. J. Sports Marketing Sponsorship 10, 7–27. doi: 10.1007/s10640-013-9664-9 30311153

[B28] De Morton N. A. (2009). The PEDro scale is a valid measure of the methodological quality of clinical trials: a demographic study. Aust. J. Physiotherapy 55, 129–133. doi: 10.1016/s0004-9514(09)70043-1 19463084

[B34] Deeks J. J. Higgins J. P. T. Altman D. G. (2019). “ Analysing data and undertaking meta-analyses,” in Cochrane Handbook for Systematic Reviews of Interventions. Eds. Higgins J. P. T. Thomas J. Chandler J. Cumpston M. Li T. Page M. J. ( Cochrane), 241–284. doi: 10.1002/9781119536604.ch10

[B35] Deng N. Soh K. G. Abdullah B. Huang D. Xiao W. Liu H. (2023). Effects of plyometric training on technical skill performance among athletes: a systematic review and meta-analysis. PloS One 18, e0288340. doi: 10.1371/journal.pone.0288340 37459333 PMC10351709

[B57] Dos' Santos T. McBurnie A. Thomas C. Comfort P. Jones P. A. (2020). Biomechanical determinants of the modified and traditional 505 change of direction speed test. J. Strength Conditioning Res. 34, 1285–1296. doi: 10.1519/JSC.0000000000003439 31868815

[B55] Dos’Santos T. Bishop C. Thomas C. Comfort P. Jones P. A. (2019). The effect of limb dominance on change of direction biomechanics: A systematic review of its importance for injury risk. Phys. Ther. Sport 37, 179–189. doi: 10.1016/j.ptsp.2019.04.005 30986764

[B56] Dos’Santos T. Thomas C. Comfort P. Jones P. A. (2018). Comparison of change of direction speed performance and asymmetries between team-sport athletes: Application of change of direction deficit. Sports 6, 174. doi: 10.3390/sports6040174 30545155 PMC6315619

[B33] Drevon D. Fursa S. R. Malcolm A. L. (2017). Intercoder reliability and validity of WebPlotDigitizer in extracting graphed data. Behav. Modification 41, 323–338. doi: 10.1177/0145445516673998 27760807

[B38] Drouzas V. Katsikas C. Zafeiridis A. Jamurtas A. Z. Bogdanis G. C. (2020). Unilateral plyometric training is superior to volume-matched bilateral training for improving strength, speed and power of lower limbs in preadolescent soccer athletes. J. Hum. Kinetics 74, 161–176. doi: 10.2478/hukin-2020-0022 33312284 PMC7706637

[B11] Duong A. Wójcicki T. R. Carnes A. J. (2022). The effects of unilateral resistance training on muscular strength, power, and measures of core stability in resistance trained individuals. Int. J. Strength Conditioning 2 (1), 145. doi: 10.47206/ijsc.v2i1.145

[B15] Escobar Hincapie A. Agudelo Velásquez C. A. Ortiz Uribe M. García Torres C. A. Rojas Jaramillo A. (2021). Unilateral and bilateral post-activation performance enhancement on jump performance and agility. Int. J. Environ. Res. Public Health 18, 10154. doi: 10.3390/ijerph181910154 34639455 PMC8508041

[B5] Farley J. B. Barrett L. M. Keogh J. W. L. Woods C. T. Milne N. . (2020). The relationship between physical fitness attributes and sports injury in female, team ball sport players: a systematic review. Sports Med. - Open 6 (1), 45. doi: 10.1186/s40798-020-00264-9 32926228 PMC7490320

[B23] Fisher J. Wallin M. (2014). Unilateral versus bilateral lower-body resistance and plyometric training for change of direction speed. J. Athl Enhanc 6, 2. doi: 10.16926/par.2023.11.06

[B2] Gabbett T. Kelly J. Pezet T. (2007). Relationship between physical fitness and playing ability in rugby league players. J. Strength Cond. Res. 21, 1126–1133. doi: 10.1519/R-20936.1 18076242

[B48] Gonzalo-Skok O. Tous-Fajardo J. Suarez-Arrones L. Arjol-Serrano J. L. Casajús J. A. Mendez-Villanueva A. (2017). Single-leg power output and between-limbs imbalances in team-sport players: Unilateral versus bilateral combined resistance training. Int. J. Sports Physiol. Perform. 12, 106–114. doi: 10.1123/ijspp.2015-0743 27140680

[B36] Higgins J. P. Thompson S. G. (2002). Quantifying heterogeneity in a meta-analysis. Stat. Med. 21, 1539–1558. doi: 10.1002/sim.1186 12111919

[B51] Kanehisa H. Miyashita M. (1983). Specificity of velocity in strength training. Eur. J. Appl. Physiol. 52, 104–106. doi: 10.1007/BF00429034 6686117

[B19] Kassiano W. Nunes J. P. Costa B. Ribeiro A. S. Loenneke J. P. Cyrino E. S. (2025). Comparison of muscle growth and dynamic strength adaptations induced by unilateral and bilateral resistance training: a systematic review and meta-analysis. Sports Med. 55, 923–936. doi: 10.1007/s40279-024-02169-z 39794667

[B13] Lee E. L. Y. Malek N. F. A. Tan K. Pratama R. S. Mohamad N. I. Md Nadzalan A. (2021). The effects of unilateral versus bilateral resistance training on bilateral deficit, unilateral and bilateral strength adaptation among trained men. Journal of Physics: Conference Series 1793 (1), 012057. doi: 10.1088/1742-6596/1793/1/012057

[B49] Li C. Wang T. Wang M. Wang W. (2023). The effect of unilateral muscle strength training on the specific physical fitness of adolescent male soccer athletes. J. Chengdu Sport Univ. 49, 91–96. doi: 10.15942/j.jcsu.2023.04.013

[B44] Li Z. Lei S. Zhang H. Wang X. Sun Y. Fan J. . (2025). Effects of unilateral and bilateral plyometrics training on basketball players’ leg power and change of direction ability. Sport Sci. Health 21 (6), 1711–1721. doi: 10.1007/s11332-025-01390-1 30311153

[B24] Liao K. F. Nassis G. Bishop C. Yang W. Bian C. Li Y. M. (2022). Effects of unilateral vs. bilateral resistance training interventions on measures of strength, jump, linear and change of direction speed: a systematic review and meta-analysis. Biol. Sport 39, 485–497. doi: 10.5114/biolsport.2022.107024 35959319 PMC9331349

[B29] Maher C. G. Sherrington C. Herbert R. D. Moseley A. M. Elkins M. (2003). Reliability of the PEDro scale for rating quality of randomized controlled trials. Phys. Ther. 83, 713–721. doi: 10.1093/ptj/83.8.713 12882612

[B14] Makaruk H. Winchester J. B. Sadowski J. Czaplicki A. Sacewicz T. (2011). Effects of unilateral and bilateral plyometric training on power and jumping ability in women. J. Strength Conditioning Res. 25, 3311–3318. doi: 10.1519/JSC.0b013e318215fa33 22076090

[B6] Malone S. Hughes B. Doran D. A. Collins K. Gabbett T. J. (2019). Can the workload–injury relationship be moderated by improved strength, speed and repeated-sprint qualities? J. Sci. Med. Sport 22, 29–34. doi: 10.1016/j.jsams.2018.01.010 30057364

[B52] Mero A. Komi P. V. Gregor R. J. (1992). Biomechanics of sprint running. Sports Med. 13, 376–392. doi: 10.2165/00007256-199213060-00002 1615256

[B25] Moher D. Liberati A. Tetzlaff J. Altman D. G. (2009). Preferred reporting items for systematic reviews and meta-analyses: the PRISMA statement. PloS Med. 6 (7), e1000097. doi: 10.1371/journal.pmed.1000097 19621072 PMC2707599

[B50] Moran J. Ramirez-Campillo R. Liew B. . (2021). Effects of bilateral and unilateral resistance training on horizontally orientated movement performance: a systematic review and meta-analysis. Sports Med. 51, 225–242. doi: 10.1007/s40279-020-01367-9 33104995

[B42] Neves T. A. Soalheiro I. Winckler C. Michalsik L. B. Ramirez-Campillo R. Guerra R. L. F. (2025). The impact of unilateral and bilateral plyometric training combined with linear sprints on physical performance in youth male elite futsal players. Int. J. Sports Physiol. Perform. 20, 1145–1151. doi: 10.1123/ijspp.2024-0538 40555411

[B59] Nimphius S. (2017). “ Training change of direction and agility,” in Advanced Strength and Conditioning. Eds. Turner A. N. Comfort P. ( Routledge), 291–309.

[B45] Núñez F. J. Santalla A. Carrasquila I. Asian J. A. Reina J. I. Suarez-Arrones L. J. (2018). The effects of unilateral and bilateral eccentric overload training on hypertrophy, muscle power and COD performance, and its determinants, in team sport players. PloS One 13, e0193841. doi: 10.1371/journal.pone.0193841 29590139 PMC5874004

[B16] Ramirez-Campillo R. Sanchez-Sanchez J. Gonzalo-Skok O. Rodríguez-Fernandez A. Carretero M. Nakamura F. Y. (2018). Specific changes in young soccer player's fitness after traditional bilateral vs. unilateral combined strength and plyometric training. Front. Physiol. 9, 265. doi: 10.3389/fphys.2018.00265 29623049 PMC5874317

[B53] Ross A. Leveritt M. Riek S. (2001). Neural influences on sprint running. Sports Med. 31, 409–425. doi: 10.2165/00007256-200131060-00002 11394561

[B37] Shi L. Lin L. (2019). The trim-and-fill method for publication bias: practical guidelines and recommendations based on a large database of meta-analyses. Medicine 98, e15987. doi: 10.1097/MD.0000000000015987 31169736 PMC6571372

[B1] Smith D. J. (2003). A framework for understanding the training process leading to elite performance. Sport. Med. 33, 1103–1126. doi: 10.2165/00007256-200333150-00003 14719980

[B17] Speirs D. E. Bennett M. A. Finn C. V. Turner A. P. (2016). Unilateral vs. bilateral squat training for strength, sprints, and agility in academy rugby players. J. Strength Conditioning Res. 30, 386–392. doi: 10.1519/JSC.0000000000001096 26200193

[B46] Stern D. Gonzalo-Skok O. Loturco I. Turner A. Bishop C. (2020). A comparison of bilateral vs. unilateral-biased strength and power training interventions on measures of physical performance in elite youth soccer players. J. Strength Conditioning Res. 34, 2105–2111. doi: 10.1519/JSC.0000000000003659 32541618

[B18] Taniguchi Y. (1997). Lateral specificity in resistance training: the effect of bilateral and unilateral training. Eur. J. Appl. Physiol. Occup. Physiol. 75, 144–150. doi: 10.1007/s004210050139 9118980

[B27] Tao M. Nassis G. P. Song Y. Yin M. Zhu C. Lyu M. . (2025). Impact of training volume settings between unilateral training and bilateral training on athletic performance: a systematic review and meta-analysis. J. Exercise Sci. Fitness 23, 291–298. doi: 10.1016/j.jesf.2025.06.003 40689312 PMC12275932

[B21] Thorpe J. L. Ebersole K. T. (2008). Unilateral balance performance in female collegiate soccer athletes. J. Strength Conditioning Res. 22, 1429–1433. doi: 10.1519/JSC.0b013e31818202db 18714247

[B4] Tingelstad L. M. Raastad T. Till K. Luteberget L. S. (2023). The development of physical characteristics in adolescent team sport athletes: a systematic review. PloS One 18, e0296181. doi: 10.1371/journal.pone.0296181 38128047 PMC10735042

[B31] Valentine J. C. Pigott T. D. Rothstein H. R. (2010). How many studies do you need? A primer on statistical power for meta-analysis. J. Educ. Behav. Stat 35, 215–247. doi: 10.3102/1076998609346961 38293548

[B12] Waller S. M. Liu W. Whitall J. (2008). Temporal and spatial control following bilateral versus unilateral training. Hum. Mov. Sci. 27, 749–758. doi: 10.1016/j.humov.2008.03.006 18639360

[B9] Wulf G. Shea C. Lewthwaite R. (2010). Motor skill learning and performance: a review of influential factors. Med. Educ. 44, 75–84. doi: 10.1111/j.1365-2923.2009.03421.x 20078758

[B3] Xiao W. Soh K. G. Wazir M. R. W. N. Talib O. Bai X. Bu T. . (2021). Effect of functional training on physical fitness among athletes: a systematic review. Front. Physiol. 12, 738878. doi: 10.3389/fphys.2021.738878 34552511 PMC8450457

[B10] Zhang W. Chen X. Xu K. Xie H. Li D. Ding S. . (2023). Effect of unilateral training and bilateral training on physical performance: a meta-analysis. Front. Physiol. 14, 1128250. doi: 10.3389/fphys.2023.1128250 37123275 PMC10133687

[B39] Zhang X. Li G. (2026). Influence of unilateral and bilateral plyometric training integrated with linear sprinting on physical performance in youth male basketball players. Sci. Rep. 16, 5236. doi: 10.1038/s41598-026-36041-z 41535349 PMC12881345

[B30] Zhang Y. Alonso-Coello P. Guyatt G. H. Yepes-Nuñez J. J. Akl E. A. Hazlewood G. . (2019). GRADE guidelines: 19. Assessing the certainty of evidence in the importance of outcomes or values and preferences—Risk of bias and indirectness. J. Clin. Epidemiol. 111, 94–104. doi: 10.1016/j.jclinepi.2018.01.013 29452223

[B26] Zhang Y. Zhuang Y. Zhang L. Sun M. (2024). The impact of eccentric training on athlete movement speed: a systematic review. Front. Physiol. 15, 1492023. doi: 10.3389/fphys.2024.1492023 39735722 PMC11671480

[B54] Zhang Z. Qu W. Peng W. Sun J. Yue J. Guan L. . (2025). Effect of unilateral and bilateral plyometric training on jumping, sprinting, and change of direction abilities: A meta-analysis. BMC Sports Sci. Med. Rehabil. 17, 97. doi: 10.1186/s13102-025-01113-6 40281589 PMC12032719

[B22] Zhang Z. Shu F. Guo H. Zhang J. Gao J. (2026). Unilateral versus bilateral resistance training for explosive jump performance, linear sprint speed, and change-of-direction ability in male basketball players: a systematic review and meta-analysis. Front. Physiol. 17, 1798477. doi: 10.3389/fphys.2026.1798477 42376604 PMC13310738

[B41] Zhao X. Turner A. P. Sproule J. Phillips S. M. (2023). The effect of unilateral and bilateral leg press training on lower body strength and power and athletic performance in adolescent rugby players. J. Hum. Kinetics 86, 235–246. doi: 10.5114/jhk/159626 37181263 PMC10170531

